# The combination of three molecular markers can be a valuable predictive tool for the prognosis of hepatocellular carcinoma patients

**DOI:** 10.1038/srep24582

**Published:** 2016-04-15

**Authors:** Sheng-Sen Chen, Kang-Kang Yu, Qing-Xia Ling, Chong Huang, Ning Li, Jian-Ming Zheng, Su-Xia Bao, Qi Cheng, Meng-Qi Zhu, Ming-Quan Chen

**Affiliations:** 1 Department of Infectious Diseases and Hepatology, Huashan Hospital, Fudan University, Shanghai 200040, China

## Abstract

Based on molecular profiling, several prognostic markers for HCC are also used in clinic, but only a few genes have been identified as useful. We collected 72 post-operative liver cancer tissue samples. Genes expression were tested by RT-PCR. Multilayer perceptron and discriminant analysis were built, and their ability to predict the prognosis of HCC patients were tested. Receiver operating characteristic (ROC) analysis was performed and multivariate analysis with Cox’s Proportional Hazard Model was used for confirming the markers’predictive efficiency for HCC patients’survival. A simple risk scoring system devised for further predicting the prognosis of liver tumor patients. Multilayer perceptron and discriminant analysis showed a very strong predictive value in evaluating liver cancer patients’prognosis. Cox multivariate regression analysis demonstrated that DUOX1, GLS2, FBP1 and age were independent risk factors for the prognosis of HCC patients after surgery. Finally, the risk scoring system revealed that patients whose total score >1 and >3 are more likely to relapse and die than patients whose total score ≤1 and ≤3. The three genes model proposed proved to be highly predictive of the HCC patients’ prognosis. Implementation of risk scoring system in clinical practice can help in evaluating survival of HCC patients after operation.

Liver cancer constitutes a major global health problem. Hepatocellular carcinoma (HCC) is the predominant form of primary liver cancer and the third leading cause of tumor-related deaths worldwide, which account for over half a million deaths annually^1^. The prevalence of HCC is diverse significantly depending on geographic region: it is most common seen in Southeast Asia and sub-Saharan Africa where the hepatitis B virus (HBV) is endemic; especially in China, HCC leads to approximately 350,000 deaths per year^2^,^3^,^4^,^5^. Although therapeutic strategies including surgical resection, radiotherapy and chemotherapy have been developed rapidly, the prognosis of patients with HCC still remains poor due to the absence of early symptoms and speedy tumor progression and invasion during the early stages^6^,^7^,^8^. Tumor occurrence, development and metastatic potential are frequently linked to the alteration of gene expression; therefore, it is imperative to identify the potential biological markers for early diagnosis, novel therapeutic strategies and prognosis prediction in patients with HCC^9^.

Dual oxidases 1 (DUOX1), is a key phenotype of NADPH-oxidases (NOXs) family, and the main function of such gene is reactive oxygen species (ROS) production^10^,^11^. DUOX1 is predominantly found in thyroid, which is involved in the synthesis of thyroid hormones^11^. It is also highly expressed in normal epithelial cells in airway, pancreas, placenta, prostate, testis, and salivary gland^10^,^12^. Glutaminase 2(GLS2) gene is located in chromosome 12, and the proteins encoded by GLS2 gens are highly expressed in normal adult liver^13^. As a mitochondrial glutaminase, GLS2 can catalyze the hydrolysis of glutamine to glutamate and it has been identified as a p53 target gene to influence the energy metabolism^14^,^15^. Fructose-1,6-bisphosphatase-1(FBP1), which catalyzes the splitting of fructose-1,6-bisphosphate (F-1,6-BP) into fructose 6-phosphate and inorganic phosphate, is a rate-limiting enzyme in gluconeogenesis^16^. Our previous investigations^17^,^18^,^19^ have shown that over expression of DUOX1, GLS2 and FBP1 could suppress the tumor growth; moreover, the epigenetic silencing of these three genes mainly via promoter hypermethylation is common in human liver cancer.

Reactive oxygen species (ROS), chemically-reactive molecules containing oxygen, including oxygen ions and peroxides, are the key mediators of cellular oxidative stress and redox dysregulation^20^. Increased ROS levels contribute to genetic instability and cancer initiation and progression^21^,^22^. Thus, Gls2 exerts the ability to suppress tumor cell growth via regulating antioxidant defense function in cells and decreasing ROS levels^21^,^22^. Paradoxically, apart from being involved in proliferative, anti-apoptotic, metastatic, and angiogenic signaling, ROS may also exert cytotoxic and proapoptotic functions that would limit tumorigenicity and malignant progression^23^,^24^. We have previously reported that the growth inhibitory effect of DUOX1 and FBP1 as liver tumor suppressor may also be mediated through enhancing the production of intracellular ROS^17^,^19^. Although, DUOX1, GLS2 and FBP1 acts as liver tumor suppressor and mechanisms about tumor inhibiting of the three genes have been studied, the specific associations between prognosis of liver patients and expression of these three genes still remains unknown. Therefore, the present study was conducted to elucidate the effect of DUOX1, GLS2 and FBP1 on clinical outcomes in human HCC.

## Materials and Methods

### Specimen cohorts

Seventy-two patients (56 males and 16 females) from Huashan Hospital (Shanghai, China) were included in this study. All the patients underwent radical hepatic resection for HCC between 2008 and 2010. The age of the patients ranged from 16 to 84 years (mean ± standard deviation [SD], 53.67 ± 12.30 years). The criteria for radicality have been published^25^. None of the patients in this study received any preoperative chemotherapy or embolization therapy. The tumor tissues and the adjacent non-tumor tissues were collected from these patients above as frozen samples. The distance between adjacent non-tumor tissue and tumor tissue boundary was 2 cm, beyond of which was regarded as distant normal tissue. The selected tumor areas had more than 80% of tumor cells as being confirmed by histology examination. Classification of tumor stages using the tumor–node–metastasis (TNM) stage according to the 7th edition of the AJCC (American Joint Committee on Cancer) cancer staging manual^26^.

We have gotten the written informed consent obtained from all patients. Experiments and procedures were in accordance with the Helsinki Declaration of 1975, and approved by the Human Ethics Committee of Shanghai Fudan University.

### Follow-up

Follow-up ended at death or June 1st, 2013, whichever came first. Follow-up imaging was performed every 3–6 months for 2 years and then every 6–12 months. According to the revised Response Evaluation Criteria in Solid Tumors (RECIST) guidelines (version 1.1)^27^, the appearance of one or more new malignant lesions on multiphase computed tomography (CT) scan or magnetic resonance (MR) imaging denotes disease progression. Disease-free survival (DFS) was defined as the time period from the date of surgery operation to the first cancer recurrence (local or distant). Overall survival (OAS) was calculated from the date of cancer resection to death or the last contact.

### RNA/DNA extraction and reverse transcription

Total RNA and genomic DNA from human tissue samples were extracted using Trizol reagent (Invitrogen) according to the manufacturer’s instructions and their concentrations were quantified by NanoDrop 1000 (Wilmington, DE., USA). A reverse transcription reaction was performed using 1 μg of total RNA with High Capacity cDNA Reverse Transcription kit (SYBR qPCR RT Mix, FSQ-101, TOYOBO).

### Quantitative real-time PCR

The mRNA levels of DUOX1, GLS2 and FBP1 were determined by real-time PCR using SYBR Green Master Mix Kit and ABI 7500 Real-Time PCR System (Applied Biosystems, Foster City, CA, USA). Glyceraldehyde-3- phosphate dehydrogenase (GAPDH) was used as an internal control of RNA integrity. The 2^−ΔΔct^ method was used to analyze the relative changes in genes expression from real-time PCR experiments^28^. Real-time PCR was performed in triplicate. We used Primer3 software to design the primers for DUOX1, GLS2, and FBP1 (primer sequences and annealing temperature are shown in Table S1).

### Statistic analysis

The differences between gene expression levels of DUOX1, GLS2 and FBP1 in diverse prognosis statuses were analyzed by Mann–Whitney U-test. Categorical variables were summed up as counts and compared by Fisher’s exact test. Biomarkers data were used to build neural network (multilayer perceptron) and to perform discriminant analysis.

Artificial neural networks (ANNs) can be employed to describe the relationship and predict the trend^29^,^30^. The most commonly used ANN in clinical practice is the multilayer perceptron (MLP)^31^. The MLP used in this study comprised one input layer with three variables (DUOX1, GLS2 and FBP1), one hidden layer with twelve neuron nodes, and one output layer with two neurons representing the predictive prognosis. This model contains 72 cases to be trained. The activation function of the hidden layer and the output layer was the hyperbolic tangent sigmoid function. The discriminant analysis is a multivariate statistical method of classification, and the classification of a case (liver tumor sample) is based on the combination of prior probabilities with discriminant functions.

In addition, we also measured the area under the curve (AUC) of the receiver operating characteristic (ROC) curve for the three genes in order to validate the predictive accuracy of our molecular computational models. Then the multivariate analysis with Cox’s proportional hazard model was performed to further confirm the authenticity and validity of the three markers’ predictive efficiency for HCC patients’ prognosis. A simple risk score devised by using significant variables obtained from Cox’s regression analysis with P < 0.05. The discrimination capability of the simple risk score was also presented by ROC curve. Finally, the Cox’s Proportional Hazard Model in which risk score included was used for predicting the hazard trends of HCC recurrence and patients’ death after surgery. All statistical tests were two-sided, and P values less than 0.05 were considered as statistically significant. The statistical analyses were performed using SPSS version 21.0 and GraphPad Prism version 5.0.

## Results

### Correlations between prognosis and clinicopathological factors in HCC patients

A total of 72 patients were enrolled during the study period. Clinical and demographic characteristics of the patients were shown in Table 1, which revealed that recurrence and death all were correlated with hepatitis B surface antigen (HBsAg) expression; interestingly, age (60 years old was taken as cutoff value according to Gokcan’s study^32^) and tumor stage were related to patients’ death only and seemed to have not any relationship with HCC recurrence; however, the differences of patients’ gender, tumor size (5 cm was considered as cutoff value according to Hwang’s study^33^), hepatitis B e antigen (HBeAg) levels, histological grade, α-fetoprotein (AFP, 100 ng/dl was identified as cutoff value based on the Brian’s research^34^) levels, intrahepatic metastasis, hepatic cirrhosis, and lesion location did not appear to have any correlation with prognosis (recurrence and death).

**Figure 1:**
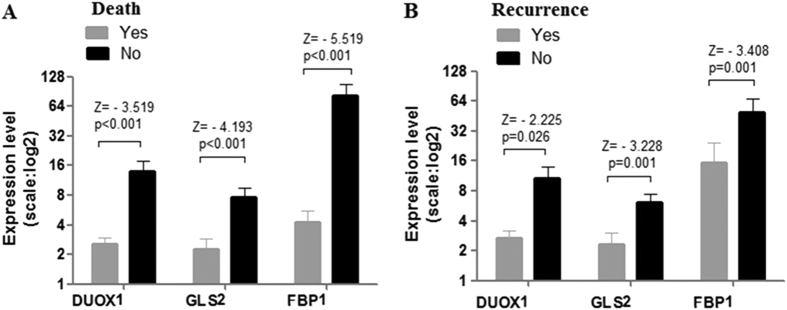
Expression levels for each marker in the groups with different prognoses. P values were identified by the Mann–Whitney U-test.

We tested DUOX1, GLS2 and FBP1 gene expression in 72 samples to better understand the relationships between their expression and prognosis status. The three markers were significantly overexpressed in non death group (DUOX1, p < 0.001; GLS2, p < 0.001; FBP1, p < 0.001) (Fig. 1A) and non recurrence group (DUOX1, p = 0.026; GLS2, p = 0.001; FBP1, p = 0.001) (Fig. 1B) compared with their counterpart groups.

### Building molecular computational models: classification of HCC patients’prognosis

In this study, gene expression data were used to build MLP and to perform discriminant analyses in order to predict the probability of recurrence and death for individual patient. The MLP classifier made up of DUOX1, GLS2 and FBP1 on 72 tumor samples, resulted in the overall predictive power of 93.1% and 86.1% respectively for overall survival (OS) and disease-free survival (DFS) (Table 2). It is interesting to note that this model correctly classified 95.6% of the samples in the death group and 82.9% of the samples in the recurrence group (Table 2). The predictive power of DUOX1, GLS2 and FBP1 expressions to discern death and recurrence from prognosis status was also confirmed by discriminant analysis that showed the overall predictive power of 86.1% and 66.7% respectively for OS and DFS (Table 3). Also, more importantly, it correctly classified 97.8% of the samples in the death group and 91.4% of the samples in the recurrence group (Table 3; Tables S2 and S3).

### ROC curve analysis

**Figure 2:**
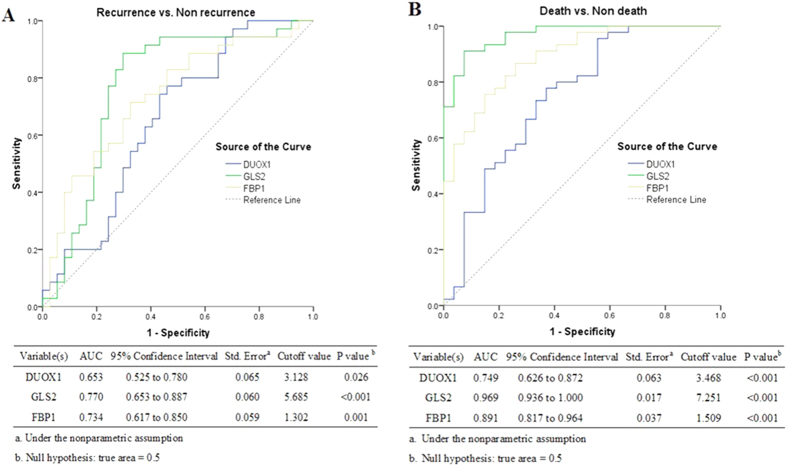
ROC analyses of DUOX1, GLS2 and FBP1 for predicting the prognosis of HCC patients (recurrence and death).

In order to determine the model robustness for predicting prognosis of HCC patient after tumor resection, we finally resorted to ROC curve analyses by individually using the expression of each marker (DUOX1, GLS2, FBP1) (Fig. 2). Among all markers, GLS2 and FBP1 showed higher AUC than DUOX1 for DFS (GLS2, AUC 0.770; FBP1, AUC 0.734; DUOX1, AUC 0.653; Fig. 2A) and for OS (GLS2, AUC 0.969; FBP1, AUC 0.891; DUOX1, AUC 0.749; Fig. 2B). The optimal cutoff values of the three genes expression levels were determined to maximize the sum of sensitivity and specificity, the detailed can be seen in Fig. 2. Therefore, patients were further categorized into two groups based on the cutoff values of the three genes expression levels (Tables S4 and S5).

### Multivariate analysis with Cox proportional hazards model

Moreover, the multivariate Cox’s proportional hazard model, in which the factors such as DUOX1, GLS2, FBP1, age, intrahepatic metastasis, histological grade, tumor stage and HBsAg were respectively included, was performed to deeply investigate the independent prognostic factors for patients’ survival. The results of the multivariable analysis showed that genes expression levels of DUOX1, GLS2 and FBP1 were significantly correlated with DFS (Table 4) and OS (Table 5). Additionally, age was also an independent risk factor for DFS (RR = 3.138, p = 0.047) and OS (RR = 3.409, p = 0.014). It’s interesting that intrahepatic metastasis was merely relevant with OS (RR = 2.905, p = 0.027, Table 5), while histological grade, tumor stage and HBsAg levels did not appear to have any associations with patients’ survival (Tables 4 and 5).

### A simple risk score for predicting the HCC patients’ prognosis

**Figure 3:**
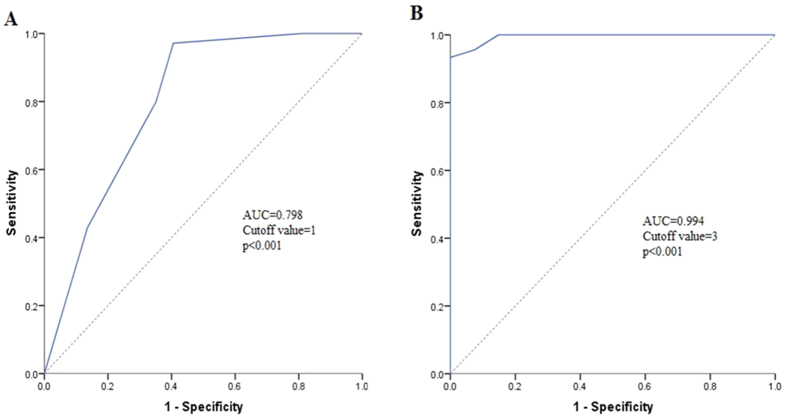
ROC curves with simplified risk score to predict the HCCs’ prognosis.

**Figure 4:**
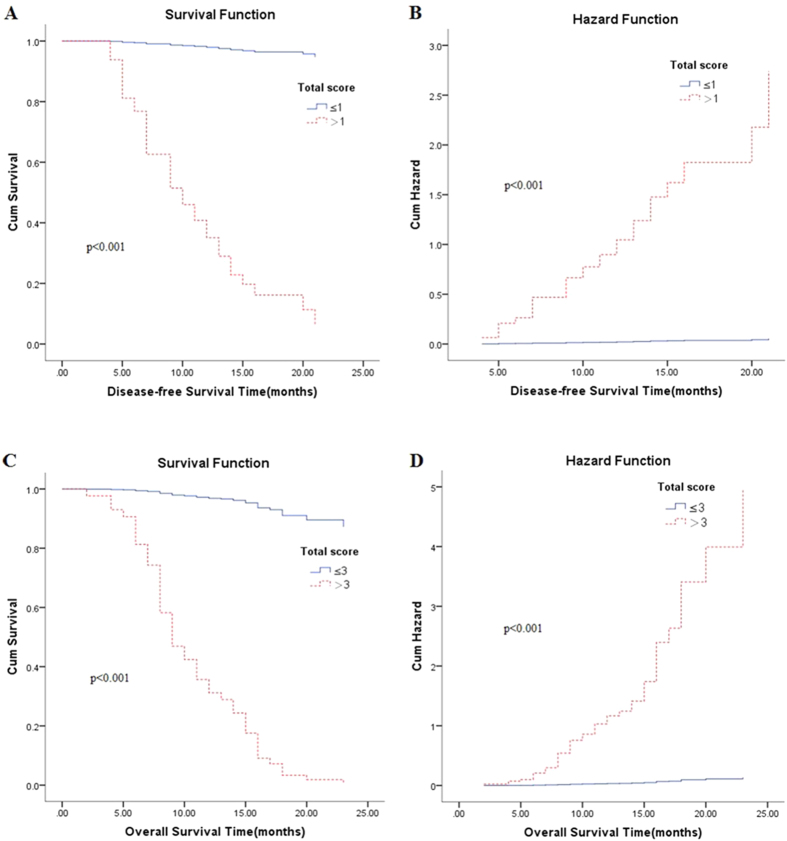
The impact of total scoring system on disease-free survival and overall survival with Cox’s regression analysis; p values were confirmed with Cox proportional hazards model.

Subsequently, a simple risk score devised by using significant variables (the independent factors both for DFS and OS: DUOX1, GLS2, FBP1 and age) in the Cox model with P < 0.05. The score was the weighted sum of those variables of which the weights were defined as the quotient (rounded to nearest integer) of corresponding estimated coefficients from a Cox’s regression analysis divided by the smallest regression coefficient in the same Cox model (Tables S6 and S7). The total score ranged from 0 to 4 was used to evaluate DFS. OAS was predicted by the total score ranged from 0 to 5. The prognosis statuses of HCC patients were taken as final diagnosis, and the total score was considered as diagnostic test. Then two ROC curves were plotted to assess the efficiency of the scoring system for predicting patients’ prognosis. Area under the curve for evaluating DFS was 0.798 (Fig. 3A) and for assessing OS was 0.994 (Fig. 3B). The optimal cutoff points of the two ROC curves were score 1(DFS prediction score, Fig. 3A) and score 3(OS prediction score, Fig. 3B) severally. For clinical and informative application, patients were further categorized into two risk groups to evaluate DFS (total score, ≤1 vs. >1) and OAS (total score, ≤3 vs. >3). From Fig. 4, we found that patients whose total score more than 1 were more likely to relapse and total score more than 3 were apt to die than patients whose score less than 1 and 3.

## Discussion

Tumor occurrence and development can be considered as the accumulation of gene mutations and epigenetic modifications. The predominant consequence of this accumulation is the activation of proto-oncogenes or silencing of tumor-suppressor genes^35^. Besides, it has been well established that cell cycle checkpoints are closely linked with tumor initiation and progression, and one of the checkpoints, G2/M checkpoint, blocks the entry into mitosis when DNA is damaged^36^. From our previous researches, liver cancer cell growth suppression induced by ectopic DUOX1, GLS2 and FBP1 expression seems to be caused by increasing G2/M phase cell number^17^,^18^,^19^, which implied that the three genes suppressed tumor cell growth through inducing G2/M phase cell cycle arrest. It is now widely accepted that constitutively elevated levels of cellular oxidative stress and dependence on mitogenic and anti-apoptotic reactive oxygen species (ROS) signaling in cancer cells are involved in the carcinogenesis^37^. GLS2 can regulate antioxidant defense function in cells by decreasing reactive oxygen species (ROS) levels and protect cells from oxidative stress that is known to contribute to genetic instability^21^,^22^. Regardless of ROS’s role in cancer initiation and progression, a recent report linked intracellular ROS accumulation to the establishment of senescence, thereby connecting ROS to tumor suppression^29^,^38^. This is in contrast to the well-described tumor-promoting activities of ROS, which have been implicated in enhanced cell proliferation and metastasis. Accordingly, our previous study^17^,^19^ also demonstrated that DUOX1 and FBP1 exerted cytotoxic and proapoptotic functions and suppressed tumorigenicity and malignant progression through enhancing the production of intracellular ROS.

In this study, we further investigate the impact of DUOX1, GLS2 and FBP1 genes expression on liver patients’prognosis after tumor resection. Medical prediction were progressing quickly as a result of computational advances, for example computation model like discriminant analysis and MLP. MLP and discriminant analysis made up of DUOX1, GLS2, and FBP1 performed on data collected from liver tumor samples showed a very strong overall predictive value for evaluating overall survival (overall predictive percent: MLP 93.1%, discriminant analysis 86.1%) and disease-free survival (overall predictive percent: MLP 86.1%, discriminant analysis 66.7%). It is noteworthy that MLP showed a correct classification of 95.6% of the samples in the death group and 82.9% in the recurrence group (Table 2), while 97.8% in death group and 91.4% in recurrence group by discriminant analysis (Table S3). Based on the discriminant analysis, the posterior probability of prognosis resulted to range between 65% and 100% for almost all disease cases, few classification errors occurred when the posterior probability was higher than 80%(Tables S2 and S3). Hence, the use of the three genes as a case classifier strengthens their importance as post-operative predictors for the prognosis of HCC patients. Using the dataset from the computational model (MLP and discriminant analysis), we also performed ROC analysis in order to optimize the model for negative and positive predictive values in patients’s prognosis. The ROC curves of GLS2 and FBP1 had a higher predictive efficiency for patients’ survival, the AUCs of the two genes to assess DFS were 0.770 and 0.734 (Fig. 2A), and to assess OS were 0.969 and 0.891(Fig. 2B); therefore, they alone and in combination can be used to evaluate survival of HCC patients. On the other hand, DUOX1 relevantly contributed to strengthening the predictive power, even if the AUC of this gen to evaluate DFS was 0.653 (Fig. 2A) and to evaluate OS was 0.749 (Fig. 2B).

Apart from the influence of DUOX1, GLS2 and FBP1 on prognosis of HCC patients, relationships between other clinical factors and prognosis was fuzzily confirmed by Fisher’s exact test, the results was summarized in Table 1, which showed that HBsAg expression was significantly correlated with recurrence (p = 0.014) and death (p = 0.034). But what’s baffling is that age and tumor stage were merely relevant with patients’ death (age, p = 0.018; tumor stage, p = 0.036) and showed no relationship with HCC recurrence (age, p = 0.076; tumor stage, p = 0.055). Additionally, gender, tumor size, HBeAg levels, histological grade, AFP levels, intrahepatic metastasis, hepatic cirrhosis, and lesion location were roughly identified as irrelevant factors for the prognosis of HCC patients after operation. Since Fisher’s exact test could hardly manage the interference existed among these variables above, a multivariate analysis must be performed to identify the authenticity and validity of the prognostic factors detected from the Table 1. According to the results of Fisher’s exact test and computational model (MLP and discriminant analysis), ultimately, Cox multivariate regression analysis was performed included the factors such as DUOX1, GLS2, FBP1, age, intrahepatic metastasis, tumor stage, histological grade and HBsAg. The data demonstrated that DUOX1, GLS2, FBP1 and age were independent risk factors for the prognosis of HCC patients after operation. As shown in Table 4, the group with DUOX1, GLS2 and FBP1 low expression may have 2.562, 2.540 and 3.529 times risk of liver cancer relapse compared with these genes high expression group. We also could observe from Table 5 that patients with DUOX1, GLS2 and FBP1 low expression were more likely to die, and the hazard ratios of death were 2.876, 2.696, and 5.170 respectively. Besides, patients with age more than 60 years have 3.138 times risk of tumor recurrence and 3.409 times risk of death compared to the counterparts with age less than 60 years. Furthermore, intrahepatic metastasis appeared to irrelevant with disease-free survival and only affected overall survival, indicating that intrahepatic metastasis and recurrence were two independent events. Finally, the Cox regression analysis suggested that tumor stage, histological grade and HBsAg seemed uncorrelated with DFS and OAS, which was conflicted with other researches’ results (the impact of tumor stage and histological grade on HCC prognosis)^39^,^40^. The reason leading to the partial difference between this research and other studies mainly may be attributed to the small sample size. In our present study, only 72 HCC patients was included, which could hide the statistical significance of some variables in the Cox regression analysis. Therefore, a large sample is needed in the future research to ensure the authenticity and stability of the results.

In order to deeply investigate the impact of DUOX1, GLS2, FBP1 and age on DFS and OAS, we developed a simple score composed of the four variables to predict the risk of HCC relapse and death after tumor resection. The cutoff values of scores were determined by ROC curve. The strong predictive power of scoring system to evaluate prognosis can be described by the ROC curves (DFS, AUC = 0.798, Fig. 2A; OS, AUC = 0.994, Fig. 2B). Patients with prediction score of ≤1 vs. >1 had distinctly different risk of HCC relapse and with total score of ≤3 vs. >3 had significantly different risk of HCC patients’ death. Notably, patients with total score ≤1 are low risk of HCC recurrence and with total score ≤3 are low risk for the death of HCC patients (Fig. 4). Identification of patients’ risk for their prognosis could initiate an individualized surveillance program for HCC patients after tumor resection.

Apart from the hepatitis B virus, hepatitis C virus (HCV) is also an important reason for HCC development^3^,^41^. Growing evidence suggests that aggressive nonalcoholic steatohepatitis (NASH) accounts for a large proportion of idiopathic or cryptogenic cirrhosis, and appears to play a significant role in the carcinogenesis of HCC^42^. Therefore, the expression of DUOX1, GLS2and FBP1 may change in the patients with hepatitis C and NASH. Furthermore, these three genes expression may also influence prognosis of the patients with hepatitis C and NASH. However, these hypotheses still need further research to be confirmed.

In conclusion, herein we were able to develop a statistical model (MLP and discriminant analysis) that accurately predict prognosis of HCC patients using a panel of three genes (DUOX1, GLS2 and FBP1). Then a multivariate analysis with Cox’s proportional hazard model manifested that DUOX1, GLS2, FBP1 and age were independent risk factors for the prognosis of HCC patients after tumor resection. These results together implied that DUOX1, GLS2 and FBP1 could well be considered as novel biomarkers for prognosis in liver cancer. What’s more, the scoring system including DUOX1, GLS2 and FBP1 acted as predictive model firstly used in our study to predict HCC patients’ survival and this predictive model can be a potential prognostic tool for liver cancer patients.

## Additional Information

**How to cite this article**: Chen, S.-S. *et al.* The combination of three molecular markers can be a valuable predictive tool for the prognosis of hepatocellular carcinoma patients. *Sci. Rep.* **6**, 24582; doi: 10.1038/srep24582 (2016).

## Supplementary Material

Supplementary Information

## Figures and Tables

**Table 1:** Demographic and clinical characteristics of the subjects enrolled in the study.

Variable	Recurrence	Non recurrence	P	Non death	Death	P
Gender						
Male(n)	27	29	0.900	20	36	0.572
Female(n)	8	8		7	9	
Age						
≥60 years(n)	28	22	0.076	14	36	0.018
<60 years(n)	7	15		13	9	
Tumor size						
≥5 cm(n)	23	26	0.802	18	31	0.845
<5 cm(n)	12	11		9	14	
Histological grade						
1 or 2(n)	27	29	0.562	21	35	0.610
3(n)	8	8		6	10	
Tumor stage						
I or II(n)	16	26	0.055	20	22	0.036
III or IV(n)	19	11		7	23	
HBsAg						
Positive(n)	34	28	0.014	20	42	0.034
Negative(n)	1	9		7	3	
HBeAg						
Positive(n)	11	12	0.927	6	17	0.201
Negative(n)	24	25		21	28	
AFP						
≥100 ng/dl(n)	20	18	0.490	16	22	0.468
<100 ng/dl(n)	15	19		11	23	
Intrahepatic metastasis						
Yes(n)	9	6	0.391	3	12	0.143
No(n)	26	31		24	33	
Hepatic cirrhosis						
Yes(n)	11	16	0.338	9	18	0.623
No(n)	24	21		18	27	

HBsAg: hepatitis B surface antigen.

HBeAg: hepatitis B e antigen.

AFP: alpha fetoprotein.

n: the sample number.

Histological grade: according to the three-tier grading scheme.

TNM stage: tumor–node–metastasis, according to the 7th edition of the AJCC (American Joint Committee on Cancer) cancer staging manual.

P value according to the Fisher exact test.

**Table 2:** Classification table of neural network (multilayer perceptron).

Actual prognosis	Group size	Predicted prognosis		
		Non death	Death	Correct percentage
Non death	27	24	3	88.9%
Death	45	2	43	95.6%
Overall percent				93.1%
		**Recurrence**	**Non recurrence**	
Recurrence	35	29	6	82.9%
Non recurrence	37	4	33	89.2%
Overall percent				86.1%

Predictive power of DUOX, GLS2, and FBP1 for predicting the prognosis of HCC patients: among the 72 cases used to train the model, the overall predictive percents were 93.1% and 86.1%.

**Table 3:** Classification table of discriminant analysis.

Actual prognosis	Group size	Predicted prognosis		
		Non death	Death	Correct percentage
Non death	27	18	9	66.7%
Death	45	1	44	97.8%
Overall percent				86.1%
		**Recurrence**	**Non recurrence**	
Recurrence	35	32	3	91.4%
Non recurrence	37	21	16	43.2%
Overall percent				66.7%

Predictive power of DUOX1, GLS2, and FBP1 for predicting the prognosis of HCC patients. This procedure is designed to develop a set of discriminating functions which can help predict survivor vs. non survivor and recurrence vs. non recurrence based on the values of other quantitative variables; 72 cases were used to develop a model to discriminate among the survivor vs. non survivor and recurrence vs. non recurrence; three predictor variables were entered. Amongst the 72 observations used to fit the model, 86.1% or 66.7% were correctly classified.

**Table 4:** Multivariate analysis of prognostic factors in patients with HCC as evaluated by disease-free survival.

Parameter	β	RR	95%CI	P
Relative DUOX1 mRNA level (<3.128 vs. ≥3.128)	0.941	2.562	1.106–5.934	0.028
Relative GLS2 mRNA level (<5.685 vs. ≥5.685)	0.932	2.540	1.061–7.479	0.041
Relative FBP1 mRNA level (<1.302 vs. ≥1.302)	1.261	3.529	1.073–8.796	0.035
Age(≥60 years vs. <60 years)	1.144	3.138	1.014–9.711	0.047
Intrahepatic metastasis(Yes vs. No)	0.821	2.273	0.759–6.807	0.142
TNM stage(III or IV vs. I or II)	0.155	1.167	0.500–2.726	0.721
Histological grade(3 vs.1 or 2)	0.498	1.646	0.658–4.119	0.287
HBsAg(Positive vs. Negative)	1.127	3.088	0.383–24.862	0.289

RR: risk ratio; 95%CI: 95% confidence interval.

β: regression coefficient of the Cox proportional hazards model.

P-value < 0.05 according to univariate Cox proportional hazards model.

Histological grade: according to the three-tier grading scheme.

TNM stage: tumor–node–metastasis, according to the 7th edition of the AJCC (American Joint Committee on Cancer) cancer staging manual.

**Table 5:** Multivariate analysis of prognostic factors in patients with HCC as evaluated by overall survival.

Parameter	β	RR	95%CI	P
Relative DUOX1 mRNA level (<3.468 vs. ≥3.468)	1.057	2.876	1.309–6.321	0.009
Relative GLS2 mRNA level (<7.251 vs. ≥7.251)	0.992	2.696	1.076–9.424	0.038
Relative FBP1 mRNA level (<1.509 vs. ≥1.509)	1.643	5.170	1.415–18.883	0.012
Age(≥60 years vs. <60 years)	1.226	3.409	1.281–9.070	0.014
Intrahepatic metastasis(Yes vs. No)	1.067	2.905	1.129–7.479	0.027
TNM stage(III or IV vs. I or II)	0.328	1.389	0.665–2.899	0.382
Histological grade(3 vs.1 or 2)	0.145	1.156	0.525–2.546	0.719
HBsAg(Positive vs. Negative)	0.368	1.431	0.382–5.464	0.588

RR: risk ratio; 95%CI: 95% confidence interval.

β: regression coefficient of the Cox proportional hazards model.

P-value < 0.05 according to univariate Cox proportional hazards model.

Histological grade: according to the three-tier grading scheme.

TNM stage: tumor–node–metastasis, according to the 7th edition of the AJCC (American Joint Committee on Cancer) cancer staging manual.
