# Supplementary material for: The combination of three molecular markers can be a valuable predictive tool for the prognosis of hepatocellular carcinoma patients
Source: Sci Rep. 2016 Apr 15;6:24582. doi: 10.1038/srep24582 (PMC4832332; doi:10.1038/srep24582)
Supplement: Supplementary Information [file srep24582-s1.pdf]

# The combination of three molecular markers can be a valuable predictive tool for the prognosis of hepatocellular carcinoma patients

Sheng-Sen Chen[1], Kang-Kang Yu[1], Qing-Xia Ling[1], Chong Huang[1], Ning Li[1], Jian-Ming Zheng[1], Su-Xia Bao[1], Qi Cheng[1], Meng-Qi Zhu[1], Ming-Quan Chen [1*]

1. Department of Infectious Diseases and Hepatology, Huashan Hospital, Fudan University, Shanghai 200040, China;

**Table S1: Set of primers used for genes analyses**

| Primers for expression study | | |
|---|---|---|
| Name | Sequence (5’-3’) | Annealing temperature(℃) |
| DUOX1 F | CCACCAGGAGTGGCATAAGT | 60 |
| DUOX1 R | CAGCTGACGGATGACTTGAA | 60 |
| GLS2 F | TCCAGCTGTGTTCTGTGGAG | 60 |
| GLS2 R | GCAAACTGGCCAGAGAAGTC | 60 |
| FBP1 F | ATCCCCTTGATGGATCTTCC | 60 |
| FBP1 R | TCCAGCATGAAGCAGTTGAC | 60 |

**Table S2: Probabilities list of discriminant analysis for HCC recurrence**

| Case | Actual Group | Highest Group | | | | | Second Highest Group | | | Discriminant Scores |
|---|---|---|---|---|---|---|---|---|---|---|
| | | Predicted Group | P(D>d \| G=g) | | P(G=g \| D=d) | Squared Distance | Group | P(G=g \| D=d) | Squared Distance | Function 1 |
| | | | p | df | | | | | | |
| 1 | 1 | 1 | 0.953 | 1 | 0.590 | 0.003 | 0 | 0.410 | 0.729 | -0.467 |
| 2 | 1 | 1 | 0.810 | 1 | 0.624 | 0.058 | 0 | 0.376 | 1.074 | -0.650 |
| 3 | 0 | 0 | 0.889 | 1 | 0.605 | 0.019 | 1 | 0.395 | 0.874 | 0.526 |
| 4 | 1 | 1 | 0.962 | 1 | 0.569 | 0.002 | 0 | 0.431 | 0.559 | -0.361 |
| 5 | 1 | 1 | 0.950 | 1 | 0.566 | 0.004 | 0 | 0.434 | 0.538 | -0.347 |
| 6 | 1 | 1 | 0.799 | 1 | 0.529 | 0.065 | 0 | 0.471 | 0.293 | -0.155 |
| 7 | 0 | 0 | 0.166 | 1 | 0.805 | 1.916 | 1 | 0.195 | 4.752 | 1.771 |
| 8 | 0 | 0 | 0.988 | 1 | 0.581 | 0.000 | 1 | 0.419 | 0.657 | 0.401 |
| 9 | 0 | 0 | 0.000 | 1 | 0.957 | 12.344 | 1 | 0.043 | 18.568 | 3.900 |
| 10 | 0 | 1** | 0.974 | 1 | 0.585 | 0.001 | 0 | 0.415 | 0.686 | -0.442 |
| 11 | 0 | 0 | 0.000 | 1 | 0.977 | 18.685 | 1 | 0.023 | 26.196 | 4.709 |
| 12 | 1 | 0** | 0.752 | 1 | 0.516 | 0.099 | 1 | 0.484 | 0.231 | 0.071 |
| 13 | 1 | 1 | 0.832 | 1 | 0.619 | 0.045 | 0 | 0.381 | 1.015 | -0.621 |
| 14 | 1 | 1 | 0.853 | 1 | 0.542 | 0.034 | 0 | 0.458 | 0.373 | -0.224 |
| 15 | 1 | 1 | 0.899 | 1 | 0.603 | 0.016 | 0 | 0.397 | 0.851 | -0.536 |
| 16 | 1 | 1 | 0.967 | 1 | 0.586 | 0.002 | 0 | 0.414 | 0.700 | -0.450 |
| 17 | 0 | 1** | 0.822 | 1 | 0.621 | 0.050 | 0 | 0.379 | 1.040 | -0.633 |
| 18 | 1 | 1 | 0.853 | 1 | 0.614 | 0.034 | 0 | 0.386 | 0.962 | -0.594 |
| 19 | 0 | 0 | 0.953 | 1 | 0.567 | 0.003 | 1 | 0.433 | 0.543 | 0.328 |
| 20 | 1 | 1 | 0.789 | 1 | 0.629 | 0.071 | 0 | 0.371 | 1.129 | -0.676 |
| 21 | 0 | 1** | 0.843 | 1 | 0.540 | 0.039 | 0 | 0.460 | 0.357 | -0.211 |
| 22 | 0 | 1** | 0.820 | 1 | 0.534 | 0.052 | 0 | 0.466 | 0.322 | -0.181 |
| 23 | 1 | 1 | 0.903 | 1 | 0.555 | 0.015 | 0 | 0.445 | 0.453 | -0.287 |
| 24 | 0 | 1** | 0.852 | 1 | 0.614 | 0.035 | 0 | 0.386 | 0.964 | -0.595 |
| 25 | 0 | 1** | 0.769 | 1 | 0.634 | 0.086 | 0 | 0.366 | 1.186 | -0.702 |
| 26 | 1 | 1 | 0.997 | 1 | 0.579 | 0.000 | 0 | 0.421 | 0.640 | -0.413 |
| 27 | 1 | 1 | 0.892 | 1 | 0.604 | 0.018 | 0 | 0.396 | 0.867 | -0.544 |
| 28 | 1 | 1 | 0.909 | 1 | 0.600 | 0.013 | 0 | 0.400 | 0.828 | -0.523 |
| 29 | 1 | 1 | 0.780 | 1 | 0.632 | 0.078 | 0 | 0.368 | 1.156 | -0.689 |
| 30 | 0 | 0 | 0.844 | 1 | 0.616 | 0.039 | 1 | 0.384 | 0.986 | 0.584 |
| 31 | 1 | 1 | 0.854 | 1 | 0.614 | 0.034 | 0 | 0.386 | 0.959 | -0.593 |
| 32 | 1 | 1 | 0.834 | 1 | 0.618 | 0.044 | 0 | 0.382 | 1.009 | -0.618 |
| 33 | 1 | 1 | 0.878 | 1 | 0.608 | 0.024 | 0 | 0.392 | 0.901 | -0.562 |
| 34 | 0 | 1** | 0.852 | 1 | 0.542 | 0.035 | 0 | 0.458 | 0.371 | -0.222 |
| 35 | 0 | 0 | 0.096 | 1 | 0.838 | 2.769 | 1 | 0.162 | 6.049 | 2.051 |
| 36 | 0 | 1** | 0.928 | 1 | 0.561 | 0.008 | 0 | 0.439 | 0.497 | -0.318 |

| | | | | | | | | | | |
|---|---|---|---|---|---|---|---|---|---|---|
| 37 | 0 | 0 | 0.977 | 1 | 0.573 | 0.001 | 1 | 0.427 | 0.587 | 0.358 |
| 38 | 0 | 1** | 0.790 | 1 | 0.629 | 0.071 | 0 | 0.371 | 1.128 | -0.675 |
| 39 | 1 | 1 | 0.849 | 1 | 0.541 | 0.036 | 0 | 0.459 | 0.366 | -0.218 |
| 40 | 0 | 0 | 0.180 | 1 | 0.799 | 1.796 | 1 | 0.201 | 4.562 | 1.727 |
| 41 | 0 | 1** | 0.780 | 1 | 0.524 | 0.078 | 0 | 0.476 | 0.267 | -0.130 |
| 42 | 0 | 0 | 0.727 | 1 | 0.510 | 0.122 | 1 | 0.490 | 0.199 | 0.037 |
| 43 | 1 | 1 | 0.869 | 1 | 0.610 | 0.027 | 0 | 0.390 | 0.922 | -0.574 |
| 44 | 0 | 1** | 0.710 | 1 | 0.505 | 0.138 | 0 | 0.495 | 0.179 | -0.037 |
| 45 | 1 | 1 | 0.844 | 1 | 0.616 | 0.039 | 0 | 0.384 | 0.986 | -0.606 |
| 46 | 1 | 1 | 0.879 | 1 | 0.608 | 0.023 | 0 | 0.392 | 0.898 | -0.561 |
| 47 | 1 | 1 | 0.808 | 1 | 0.625 | 0.059 | 0 | 0.375 | 1.079 | -0.652 |
| 48 | 0 | 1** | 0.753 | 1 | 0.517 | 0.099 | 0 | 0.483 | 0.232 | -0.095 |
| 49 | 0 | 1** | 0.811 | 1 | 0.624 | 0.057 | 0 | 0.376 | 1.070 | -0.648 |
| 50 | 0 | 1** | 0.938 | 1 | 0.563 | 0.006 | 0 | 0.437 | 0.515 | -0.331 |
| 51 | 0 | 1** | 0.780 | 1 | 0.631 | 0.078 | 0 | 0.369 | 1.155 | -0.688 |
| 52 | 0 | 1** | 0.790 | 1 | 0.629 | 0.071 | 0 | 0.371 | 1.128 | -0.675 |
| 53 | 0 | 1** | 0.860 | 1 | 0.612 | 0.031 | 0 | 0.388 | 0.944 | -0.585 |
| 54 | 1 | 1 | 0.843 | 1 | 0.540 | 0.039 | 0 | 0.460 | 0.358 | -0.211 |
| 55 | 0 | 0 | 0.948 | 1 | 0.566 | 0.004 | 1 | 0.434 | 0.534 | 0.322 |
| 56 | 1 | 1 | 0.830 | 1 | 0.620 | 0.046 | 0 | 0.380 | 1.021 | -0.624 |
| 57 | 1 | 1 | 0.767 | 1 | 0.635 | 0.088 | 0 | 0.365 | 1.193 | -0.705 |
| 58 | 0 | 1** | 0.950 | 1 | 0.591 | 0.004 | 0 | 0.409 | 0.737 | -0.472 |
| 59 | 0 | 0 | 0.872 | 1 | 0.547 | 0.026 | 1 | 0.453 | 0.402 | 0.225 |
| 60 | 1 | 1 | 0.888 | 1 | 0.605 | 0.020 | 0 | 0.395 | 0.877 | -0.550 |
| 61 | 1 | 0** | 0.778 | 1 | 0.632 | 0.079 | 1 | 0.368 | 1.161 | 0.669 |
| 62 | 0 | 1** | 0.954 | 1 | 0.567 | 0.003 | 0 | 0.433 | 0.545 | -0.351 |
| 63 | 1 | 1 | 0.794 | 1 | 0.628 | 0.068 | 0 | 0.372 | 1.118 | -0.670 |
| 64 | 1 | 0** | 0.336 | 1 | 0.747 | 0.926 | 1 | 0.253 | 3.090 | 1.349 |
| 65 | 1 | 1 | 0.931 | 1 | 0.562 | 0.007 | 0 | 0.438 | 0.503 | -0.323 |
| 66 | 1 | 1 | 0.769 | 1 | 0.634 | 0.087 | 0 | 0.366 | 1.188 | -0.703 |
| 67 | 0 | 1** | 0.940 | 1 | 0.564 | 0.006 | 0 | 0.436 | 0.518 | -0.333 |
| 68 | 0 | 1** | 0.987 | 1 | 0.582 | 0.000 | 0 | 0.418 | 0.659 | -0.425 |
| 69 | 1 | 1 | 0.777 | 1 | 0.632 | 0.080 | 0 | 0.368 | 1.163 | -0.692 |
| 70 | 0 | 0 | 0.001 | 1 | 0.946 | 10.176 | 1 | 0.054 | 15.884 | 3.577 |
| 71 | 0 | 0 | 0.188 | 1 | 0.797 | 1.737 | 1 | 0.203 | 4.467 | 1.705 |
| 72 | 0 | 0 | 0.654 | 1 | 0.662 | 0.201 | 1 | 0.338 | 1.547 | 0.835 |
| **. Misclassified case 0=non recurrence, 1=recurrence | | | | | | | | | | |

The classification table lists the two highest groups amongst the classification functions for each of the 72 observations used to fit the model, as well as for any new observations. P (G | D) in the discriminant analysis was identified as posterior probability.

**Table S3: Probabilities list of discriminant analysis for the death of HCC patients**

| Case | Actual Group | Highest Group | | | | | Second Highest Group | | | Discriminant Scores |
|---|---|---|---|---|---|---|---|---|---|---|
| | | Predicted Group | P(D>d \| G=g) | | P(G=g \| D=d) | Squared Distance | Group | P(G=g \| D=d) | Squared Distance | Function 1 |
| | | | p | df | | | | | | |
| 1 | 1 | 1 | 0.847 | 1 | 0.752 | 0.037 | 0 | 0.248 | 2.261 | -0.443 |
| 2 | 1 | 1 | 0.959 | 1 | 0.795 | 0.003 | 0 | 0.205 | 2.709 | -0.585 |
| 3 | 0 | 0 | 0.705 | 1 | 0.690 | 0.143 | 1 | 0.310 | 1.740 | 0.683 |
| 4 | 1 | 1 | 0.718 | 1 | 0.696 | 0.130 | 0 | 0.304 | 1.785 | -0.275 |
| 5 | 1 | 1 | 0.718 | 1 | 0.696 | 0.131 | 0 | 0.304 | 1.784 | -0.275 |
| 6 | 1 | 1 | 0.681 | 1 | 0.678 | 0.169 | 0 | 0.322 | 1.654 | -0.225 |
| 7 | 0 | 0 | 0.577 | 1 | 0.916 | 0.311 | 1 | 0.084 | 5.085 | 1.619 |
| 8 | 0 | 0 | 0.697 | 1 | 0.686 | 0.152 | 1 | 0.314 | 1.710 | 0.671 |
| 9 | 0 | 0 | 0.216 | 1 | 0.972 | 1.530 | 1 | 0.028 | 8.609 | 2.298 |
| 10 | 1 | 1 | 0.808 | 1 | 0.736 | 0.059 | 0 | 0.264 | 2.115 | -0.393 |
| 11 | 0 | 0 | 0.000 | 1 | 1.000 | 17.674 | 1 | 0.000 | 34.824 | 5.265 |
| 12 | 1 | 0** | 0.419 | 1 | 0.517 | 0.653 | 1 | 0.483 | .790 | 0.253 |
| 13 | 1 | 1 | 0.969 | 1 | 0.819 | 0.002 | 0 | 0.181 | 3.014 | -0.675 |
| 14 | 1 | 1 | 0.685 | 1 | 0.679 | 0.165 | 0 | 0.321 | 1.667 | -0.230 |
| 15 | 1 | 1 | 0.881 | 1 | 0.766 | 0.022 | 0 | 0.234 | 2.395 | -0.487 |
| 16 | 1 | 1 | 0.735 | 1 | 0.882 | 0.114 | 0 | 0.118 | 4.143 | -0.975 |
| 17 | 1 | 1 | 0.999 | 1 | 0.808 | 0.000 | 0 | 0.192 | 2.877 | -0.635 |
| 18 | 1 | 1 | 0.944 | 1 | 0.789 | 0.005 | 0 | 0.211 | 2.647 | -0.566 |
| 19 | 0 | 0 | 0.488 | 1 | 0.565 | 0.481 | 1 | 0.435 | 1.007 | 0.367 |
| 20 | 1 | 1 | 0.956 | 1 | 0.823 | 0.003 | 0 | 0.177 | 3.070 | -0.691 |
| 21 | 1 | 1 | 0.847 | 1 | 0.753 | 0.037 | 0 | 0.247 | 2.264 | -0.444 |
| 22 | 1 | 1 | 0.391 | 1 | 0.948 | 0.737 | 0 | 0.052 | 6.532 | -1.495 |
| 23 | 1 | 1 | 0.658 | 1 | 0.666 | 0.196 | 0 | 0.334 | 1.574 | -0.194 |
| 24 | 1 | 1 | 0.937 | 1 | 0.828 | 0.006 | 0 | 0.172 | 3.156 | -0.716 |
| 25 | 1 | 1 | 0.942 | 1 | 0.827 | 0.005 | 0 | 0.173 | 3.131 | -0.709 |
| 26 | 1 | 1 | 0.712 | 1 | 0.888 | 0.136 | 0 | 0.112 | 4.268 | -1.005 |
| 27 | 1 | 1 | 0.967 | 1 | 0.797 | 0.002 | 0 | 0.203 | 2.743 | -0.595 |
| 28 | 1 | 1 | 0.907 | 1 | 0.776 | 0.014 | 0 | 0.224 | 2.496 | -0.519 |
| 29 | 1 | 1 | 0.945 | 1 | 0.826 | 0.005 | 0 | 0.174 | 3.120 | -0.706 |
| 30 | 0 | 1** | 0.517 | 1 | 0.585 | 0.419 | 0 | 0.415 | 1.102 | 0.011 |
| 31 | 0 | 1** | 0.900 | 1 | 0.773 | 0.016 | 0 | 0.227 | 2.468 | -0.510 |
| 32 | 1 | 1 | 0.919 | 1 | 0.834 | 0.010 | 0 | 0.166 | 3.236 | -0.738 |
| 33 | 1 | 1 | 0.971 | 1 | 0.799 | 0.001 | 0 | 0.201 | 2.760 | -0.600 |
| 34 | 0 | 0 | 0.494 | 1 | 0.569 | 0.468 | 1 | 0.431 | 1.026 | 0.376 |
| 35 | 0 | 0 | 0.249 | 1 | 0.968 | 1.329 | 1 | 0.032 | 8.123 | 2.214 |

| | | | | | | | | | | |
|---|---|---|---|---|---|---|---|---|---|---|
| 36 | 1 | 1 | 0.819 | 1 | 0.862 | 0.053 | 0 | 0.138 | 3.712 | -0.866 |
| 37 | 1 | 1 | 0.314 | 1 | 0.959 | 1.013 | 0 | 0.041 | 7.309 | -1.643 |
| 38 | 1 | 1 | 0.925 | 1 | 0.832 | 0.009 | 0 | 0.168 | 3.206 | -0.730 |
| 39 | 1 | 1 | 0.450 | 1 | 0.938 | 0.572 | 0 | 0.062 | 6.019 | -1.393 |
| 40 | 0 | 0 | 0.091 | 1 | 0.987 | 2.852 | 1 | 0.013 | 11.465 | 2.750 |
| 41 | 0 | 1** | 0.685 | 1 | 0.680 | 0.165 | 0 | 0.320 | 1.668 | -0.231 |
| 42 | 0 | 0 | 0.453 | 1 | 0.541 | 0.563 | 1 | 0.459 | .896 | 0.310 |
| 43 | 1 | 1 | 0.966 | 1 | 0.820 | 0.002 | 0 | 0.180 | 3.028 | -0.679 |
| 44 | 0 | 0 | 0.535 | 1 | 0.596 | 0.385 | 1 | 0.404 | 1.159 | 0.440 |
| 45 | 1 | 1 | 0.951 | 1 | 0.792 | 0.004 | 0 | 0.208 | 2.677 | -0.575 |
| 46 | 1 | 1 | 0.934 | 1 | 0.786 | 0.007 | 0 | 0.214 | 2.606 | -0.553 |
| 47 | 1 | 1 | 0.976 | 1 | 0.816 | 0.001 | 0 | 0.184 | 2.981 | -0.666 |
| 48 | 0 | 0 | 0.563 | 1 | 0.613 | 0.334 | 1 | 0.387 | 1.252 | 0.482 |
| 49 | 1 | 1 | 0.984 | 1 | 0.803 | 0.000 | 0 | 0.197 | 2.811 | -0.616 |
| 50 | 0 | 1** | 0.665 | 1 | 0.670 | 0.187 | 0 | 0.330 | 1.599 | -0.204 |
| 51 | 1 | 1 | 0.950 | 1 | 0.824 | 0.004 | 0 | 0.176 | 3.098 | -0.699 |
| 52 | 1 | 1 | 0.978 | 1 | 0.816 | 0.001 | 0 | 0.184 | 2.975 | -0.664 |
| 53 | 1 | 1 | 0.931 | 1 | 0.785 | 0.008 | 0 | 0.215 | 2.593 | -0.550 |
| 54 | 1 | 1 | 0.757 | 1 | 0.877 | 0.096 | 0 | 0.123 | 4.025 | -0.946 |
| 55 | 0 | 0 | 0.674 | 1 | 0.674 | 0.177 | 1 | 0.326 | 1.628 | 0.640 |
| 56 | 1 | 1 | 0.970 | 1 | 0.798 | 0.001 | 0 | 0.202 | 2.754 | -0.599 |
| 57 | 1 | 1 | 0.948 | 1 | 0.825 | 0.004 | 0 | 0.175 | 3.106 | -0.702 |
| 58 | 1 | 1 | 0.875 | 1 | 0.764 | 0.025 | 0 | 0.236 | 2.372 | -0.479 |
| 59 | 0 | 1** | 0.861 | 1 | 0.758 | 0.031 | 0 | 0.242 | 2.315 | -0.461 |
| 60 | 0 | 1** | 0.700 | 1 | 0.687 | 0.149 | 0 | 0.313 | 1.719 | -0.250 |
| 61 | 0 | 0 | 0.866 | 1 | 0.849 | 0.029 | 1 | 0.151 | 3.483 | 1.230 |
| 62 | 0 | 1** | 0.620 | 1 | 0.645 | 0.246 | 0 | 0.355 | 1.442 | -0.140 |
| 63 | 1 | 1 | 0.981 | 1 | 0.815 | 0.001 | 0 | 0.185 | 2.964 | -0.661 |
| 64 | 0 | 0 | 0.481 | 1 | 0.933 | 0.498 | 1 | 0.067 | 5.773 | 1.766 |
| 65 | 1 | 1 | 0.705 | 1 | 0.689 | 0.144 | 0 | 0.311 | 1.738 | -0.258 |
| 66 | 1 | 1 | 0.923 | 1 | 0.833 | 0.009 | 0 | 0.167 | 3.219 | -0.734 |
| 67 | 0 | 1** | 0.623 | 1 | 0.647 | 0.241 | 0 | 0.353 | 1.455 | -0.145 |
| 68 | 0 | 1** | 0.741 | 1 | 0.707 | 0.109 | 0 | 0.293 | 1.867 | -0.306 |
| 69 | 1 | 1 | 0.949 | 1 | 0.825 | 0.004 | 0 | 0.175 | 3.103 | -0.701 |
| 70 | 0 | 0 | 0.000 | 1 | 1.000 | 19.645 | 1 | 0.000 | 37.570 | 5.493 |
| 71 | 0 | 0 | 0.092 | 1 | 0.987 | 2.840 | 1 | 0.013 | 11.440 | 2.746 |
| 72 | 0 | 0 | 0.641 | 1 | 0.903 | 0.217 | 1 | 0.097 | 4.678 | 1.526 |
| **. Misclassified case 0=non death, 1=death | | | | | | | | | | |

The classification table lists the two highest groups amongst the classification functions for each of the 72 observations used to fit the model, as well as for any new observations. P (G | D) in the discriminant analysis was identified as posterior probability.

**Table S4. Patients were categorized into two groups to evaluate DFS (groups depended on the optimal cutoff values of genes expression levels)**

| | Patients group | |
|---|---|---|
| Gene | Gene high expression | Gene low expression |
| DUOX1 | ≥3.128 | <3.128 |
| GLS2 | ≥5.685 | <5.685 |
| FBP1 | ≥1.302 | <1.302 |

DFS, disease-free survival

**Table S5. Patients were categorized into two groups to evaluate OS (groups depended on the optimal cutoff values of genes expression levels)**

| | Patients group | |
|---|---|---|
| Gene | Gene high expression | Gene low expression |
| DUOX1 | ≥3.468 | <3.468 |
| GLS2 | ≥7.251 | <7.251 |
| FBP1 | ≥1.509 | <1.509 |

OS, overall survival

**Table S6. Components of the disease-free survival prediction score**

| Factors | Score(rounded to nearest integer) | Score origin |
|---|---|---|
| Relative DUOX1 mRNA level | | |
| ≥3.128 | 0 | |
| <3.128 | 1 | 0.941/0.932 |
| Relative GLS2 mRNA level | | |
| ≥5.685 | 0 | |
| <5.685 | 1 | 0.932/0.932 |
| Relative FBP1 mRNA level | | |
| ≥1.302 | 0 | |
| <1.302 | 1 | 1.261/0.932 |
| Age | | |
| <60years | 0 | |
| ≥60years | 1 | 1.144/0.932 |

**Table S7. Components of the overall survival prediction score**

| Factors | Score(rounded to nearest integer) | Score origin |
|---|---|---|
| Relative DUOX1 mRNA level | | |
| ≥3.468 | 0 | |
| <3.468 | 1 | 1.057/0.992 |
| Relative GLS2 mRNA level | | |
| ≥7.251 | 0 | |
| <7.251 | 1 | 0.992/0.992 |
| Relative FBP1 mRNA level | | |
| ≥1.509 | 0 | |
| <1.509 | 2 | 1.643/0.992 |
| Age | | |
| <60years | 0 | |
| ≥60years | 1 | 1.226/0.992 |
